# Public health messaging and community engagement during COVID-19: a rapid-review from the Greater Mekong Subregion

**DOI:** 10.1186/s42522-025-00183-3

**Published:** 2025-12-08

**Authors:** Kaia Vedlog Kveen, Kyra Lilier, Marte Karoline Råberg Kjøllesdal, Mijail Naranjo-Zolotov, Patcharin Lapanun, Inês Veiga, Sara Dias, Hans Jørgen Overgaard, Kate Bärnighausen

**Affiliations:** 1 Faculty of Landscape and Society, Norwegian University of Life Science, Ås, Norway; 2 Heidelberg Institute of Global Health (HIGH), Faculty of Medicine and University Hospital, Heidelberg University, 69120 Heidelberg, Germany; 3 NOVA Information Management School (NOVA IMS), Universidade NOVA de Lisboa, Campus de Campolide, Lisboa, 1070-312 Portugal; 4 Faculty of Medicine and Faculty of Humanities and Social Sciences, Khon Kaen University, Khon Kaen, Thailand; 5 School of Health Sciences, Polytechnic of Leiria, Leiria, Portugal; 6 Wits School of Public Health, University of the Witwatersrand, Johannesburg, South Africa

**Keywords:** Rapid review, Messaging, Community engagement, Public health, Pandemics

## Abstract

**Background:**

The COVID-19 pandemic highlighted the importance of public health messaging and community engagement in reducing disease transmission. This rapid review analyzes these approaches in the Greater Mekong Subregion (GMS), a hotspot for emerging infectious diseases, to help inform future pandemic preparedness and response strategies.

**Methods:**

This rapid review follows the Preferred Reporting Items for Systematic Reviews and Meta-Analysis Checklist. We used Web of Science and PubMed databases. Articles were included if they addressed COVID-19-related public health messaging and/or community engagement initiatives, focused on countries within the GMS, were published in English between 2020 and 2024, and provided full-text access. Articles focusing on unrelated topics, such as vaccine development or adverse effects of the pandemic were excluded. Data extraction was performed using a calibrated data extraction sheet, with two researchers extracting and verifying the data.

**Results:**

After the screening process, 26 articles were included, and 24 were excluded. Three articles use qualitative methods, five articles use quantitative approaches, eleven articles are identified as descriptive and seven are literature reviews. In most countries the government employed a centralized strategy for streamlined and coherent communication using traditional media, social media and mobile applications. Vietnam demonstrated an innovative and inclusive approach to risk communication, leveraging creative approaches such as songs and slogans to disseminate messages. Thailand effectively utilized its pre-existing network of village health volunteers to inform community members, though marginalized groups remained hard to reach.

**Conclusion:**

The GMS employed diverse public health messaging and community engagement strategies during COVID-19. Our findings emphasize the importance of adaptable and inclusive strategies to ensure equitable public health outcomes in future pandemics.

## Introduction

The COVID-19 pandemic emphasized the role of public health messaging and community engagement in managing health crises, particularly in the absence of pharmaceutical interventions such as vaccines or effective treatments [1]. Early in the pandemic, non-pharmaceutical measures such as risk communication and community engagement (RCCE) were pivotal in controlling disease spread [2]. RCCE involves disseminating timely, accurate, and culturally relevant health information to empower communities to take preventive actions [2]. It also enables active participation in public health efforts, recognizing that successful health emergency management begins and ends within communities [3]. The World Health Organization (WHO) has consistently emphasized the importance of RCCE in health emergencies, identifying RCCE as one of the eight pillars of pandemic preparedness and response [4]. Lessons from past outbreaks, including SARS, MERS, and Ebola, highlight the necessity of clear, actionable, and locally relevant communication strategies to build trust and drive collective action. The GMS—comprising Cambodia, Laos, Myanmar, Thailand, Vietnam, and China’s Yunnan Province and Guangxi Zhuang Region—presents a unique context for examining RCCE strategies. As a hotspot for emerging infectious diseases and zoonotic spillovers due to high biodiversity, rapid urbanization, and extensive human-animal interactions, the region faces a heightened risk for future pandemics [5]. The diverse sociocultural landscapes and varying levels of economic development provide lessons for adapting RCCE approaches to different contexts [5]. The GMS is characterized by collectivist cultural traditions, where communal values significantly influence health behaviors, as well as disparities in press freedom and access to health resources, which can shape public health communication strategies [6].

Despite Southeast Asia’s recognized success in containing the pandemic—often attributed to prompt and stringent public health measures—comprehensive evidence on RCCE initiatives in the GMS remains limited [6, 7]. The implementation of RCCE strategies has been uneven across countries with challenges such as misinformation, infodemics, mistrust in governments, and resistance rooted in cultural or religious beliefs impeding the effectiveness of RCCE in various settings [8]. Studies on RCCE during COVID-19 in other regions, such as Africa, have identified key strategies for RCCE as capacity building, robust risk communication systems, and misinformation countermeasures [9].

This rapid review aims to address the gap in RCCE evidence by synthesizing public health messaging and community engagement initiatives employed during the COVID-19 pandemic across the GMS. We focus on how RCCE strategies were tailored to local contexts, the challenges encountered, and the lessons learned. By focusing on this region, the review will contribute to a better understanding of how cultural, socioeconomic, and structural factors influence the success of RCCE in mitigating health emergencies. 

## Methods

We followed the Preferred Reporting Items for Systematic Reviews and Meta-Analysis (PRISMA) Checklist, as recommended by Cochrane [10]. We used EndNote to manage the articles from the literature search and to upload, find duplicates, and sort the different articles.

### Greater Mekong Subregion

The GMS is a region in southeast Asia, and includes Vietnam, Thailand, Laos, Cambodia, Myanmar, and two provinces of China; Yunnan and Guangxi [11]. GMS is often referred to as a subregional economic cooperation collaboration designed to enhance economic relations [11]. The program also prioritizes subregional projects in human health. These countries’ locations make them at the frontline of potentially new disease emergencies, as the risk for disease spillovers from animals to humans, similar to COVID-19, is high in the area [12]. The region of GMS consists of countries with different levels of development, such as Laos, Cambodia, and Myanmar, which are on the UN list of the least developed countries in the world [13]. Vietnam is a lower-middle-income country [14] and Thailand is an upper-middle-income country [15]. Guanxi and Yunnan are among the least developed provinces in China [16]. The country’s development status could affect the number of academic publications made in the different countries, which are shown in Table 1. The data from this table is collected from the SCImago Journal and Country rank and includes the total number of publications published in all subject areas from 2020 to 2023.

**Table 1:** Publication numbers and the countries in the Greater Mekong Subregion

Country	Thailand	Vietnam	Myanmar	Cambodia	Laos	China	Total
Total publications	103 288	74 137	3 190	2 885	1 381	3 749 329	3 934 210
Percentage (%)	2,63	1,88	0,08	0,07	0,04	95,3	100

Source: SCImago [15]

### Eligibility criteria

Before defining relevant terms to include in the search string, inclusion and exclusion criteria were developed based on the research question.

### Inclusion criteria

- The article should relate to the COVID-19 pandemic.
- The article should include studies/reports from initiatives using community engagement strategies and/or public health messaging.
- English language.
- Publications written from 2020 to 2024.
- The results are from the countries in the Greater Mekong subregion (China in Yunnan Province and Guangxi Zhuang Region, Myanmar, Thailand, Laos, Vietnam, and Cambodia).
- Full text available.

### Exclusion criteria

- Grey literature.
- Articles not from GMS.
- Article does not mention community engagement strategies nor public health messaging.
- Article does mention community engagement strategies or public health messaging, but too superficial.
- No full text available.
- Articles focusing on the consequences of the pandemic.
- Articles focusing on vaccine development.

“Peer-review” was not included as an inclusion criterion, as many articles were published as pre-prints during the pandemic. The decision was made to ensure relevant articles were included if relevant to the research question. As the primary aim of the study was to describe RCCE strategies rather than to document the broader social, economic, or psychological consequences of the pandemic, two exclusion criteria were developed to clarify what the search should not include: (1) the consequences of the COVID-19 pandemic and (2) vaccine development. We excluded articles that focused primarily on the consequences of the pandemic, such as the social, economic, or health-related impacts of COVID-19. This decision was made to maintain a clear focus on RCCE strategies themselves, rather than the wide-ranging outcomes of the pandemic more broadly. Including such studies would have shifted attention away from how communication and engagement approaches were designed and implemented, and towards their downstream consequences, which, while important, lie beyond the scope of our review. To ensure inter-rater reliability and to reduce selection and extraction bias, two reviewers independently screened and coded the articles against the inclusion criteria. Coding discrepancies were discussed in meetings, where differences in perception were resolved through dialogue. Where disagreements persisted, we invited a third reviewer to adjudicate.

### Choice of databases

We chose Web of Science and PubMed.

### PICO-scheme and test searches

We developed a PICO scheme to organize the work of developing the search string [17]. A summary is provided in Table 2 and the complete scheme is provided in Table 3. The scheme included the headings “Population,” “Intervention,” and “Context.” PubMed and Web of Science have different setups for the database search features, resulting in two slightly different search strings that best suit the database. We included Mesh Terms in the search string for PubMed, where this was available. Mesh terms detect articles more broadly. PubMed and Web of Science accept proximity operators, which make it possible for words to be detected in the search even though they do not appear as in a phrase. One example of the use of proximity operators was (“Community-based participatory strategies“[tiab:~5]) used in PubMed and (“Community-based participatory” NEAR/5 (“strateg*” OR “approach”) used in Web of Science. Similar for both search strings was the use of truncation to include different endings of the terms in the search string. An example is the word communication, written communicat* in the search string to include various word endings. PubMed is limited to truncation; words shorter than four letters must be written as free text. After defining the terms used in the search string during the test search, we used Boolean operators (AND/OR) to build the search string. Synonyms and terminology for population, context, and intervention were combined with OR and AND between the three components of the PICO scheme. After working on the test search, we double-checked the spelling and setup of the string. This resulted in the final search strings we used on 24.01.2024. The following paragraphs describe the process of defining words for the PICO in detail.

**Table 2:** PICO (Populationl, Intervention, Context) scheme summary developing search string

Population	Intervention	Context
“Mekong” “Yunnan Province” Guangxi Thailand Laos Cambodia* Vietnam* Myanma* Burma Thai Thais Loatian* “Greater Mekong Subregion” GMS Burmese	“Communit* engagement” “Risk communicat*” “RCCE” “communicable disease control” “Health communicat*” “Public health messag*” “Community-level respon*” “Health Promotion” “PLA” “Participatory learning and action” “SBCC” “Social and behavior change communicat*” “Social mobilization” “Communit* participation” “Communit* engagement intervention*”	“Covid-19” “2019-nCoV” “sars-cov-2” “Pandemic*” “corona*” “COVID 19 Pandemic”

**Table 3:** Full search string conducted on the 24.01.2024

Database	Full search string	Filters applied
Web of Science	(Mekong OR “Yunnan Province” OR Guangxi OR Thai OR Thais OR Thailand OR Laos OR Laotian* OR Cambodia* OR Vietnam* OR Myanma* OR Burma OR Burmese OR “Greater Mekong Subregion” OR “GMS”) AND (“Communit* engagement” OR “Risk communicat*” OR RCCE OR “communicable disease control” OR “Health communicat*” OR “Public health messag*” OR “Community-level respon*” OR “Health Promotion” OR PLA OR “Participatory learning and action” OR SBCC OR “Social and behavior change communicat*” OR “Social mobilization” OR (“Risk communicat*” NEAR/3 (“community” OR “engagement”)) OR (“Communit* engagement” NEAR/5 (strateg*)) OR (“Health promot*” NEAR/5 (strateg*)) OR “Communit* participation” OR “Communit* engagement intervention*” OR (“Community-based participatory” NEAR/5 (“strateg*” OR “approach”))) AND (“Covid-19” OR “2019-nCoV” OR “sars-cov-2” OR “Pandemic*” OR “corona*” OR “COVID 19 Pandemic”)	2020–2024
PubMed	(Mekong[tiab] OR “Yunnan Province“[tiab] OR Guangxi[tiab] OR Thai[tiab] OR Thais[tiab] OR Thailand[tiab] OR Laos[tiab] OR Laotian*[tiab] OR Cambodia*[tiab] OR Vietnam*[tiab] OR Myanma*[tiab] OR Burma[tiab] OR Burmese[tiab] OR “Greater Mekong Subregion“[tiab] OR “GMS“[tiab] OR “Mekong Valley“[Mesh] OR “Thailand“[Mesh] OR “Laos“[Mesh] OR “Cambodia“[Mesh] OR “Vietnam“[Mesh] OR “Myanmar“[Mesh]) AND (“Community engagement“[tiab] OR “Risk communicat*“[tiab] OR “RCCE“[tiab] OR “communicable disease control“[tiab] OR “Communicable Disease Control“[Mesh] OR “Health communicat*“[tiab] OR “Health Communication“[Mesh] OR “Public health messag*“[tiab] OR “Community-level respon*“[tiab] OR “Health Promotion“[tiab] OR “Health Promotion“[Mesh] OR “PLA“[tiab] OR “Participatory learning and action“[tiab] OR “SBCC“[tiab] OR “Social and behavior change communicat*“[tiab] OR “Social mobilization“[tiab] OR “community engagement strategy“[tiab:~5] OR “community engagement strategies“[tiab:~5] OR “Health promotion strategy“[tiab:~5] OR “Health promotion strategies“[tiab:~5] OR “Community participation“[tiab] OR “Community Participation“[Mesh] OR “community participations“[tiab:~5] OR “Community engagement intervention“[tiab:~5] OR “Community engagement interventions“[tiab:~5] OR “Community-based participatory strategy“[tiab:~5] OR “Community-based participatory strategies“[tiab:~5] OR “Community-based participatory approach“[tiab:~5]) AND (“Covid-19” OR “COVID-19“[Mesh] OR “2019-nCoV“[tiab] OR “sars-cov-2“[tiab] OR “SARS-CoV-2“[Mesh] OR “Pandemic*“[tiab] OR “corona*“[tiab] OR “COVID 19 Pandemic“[tiab])	Full text, 2020–2024

### Screening

**Fig. 1:**
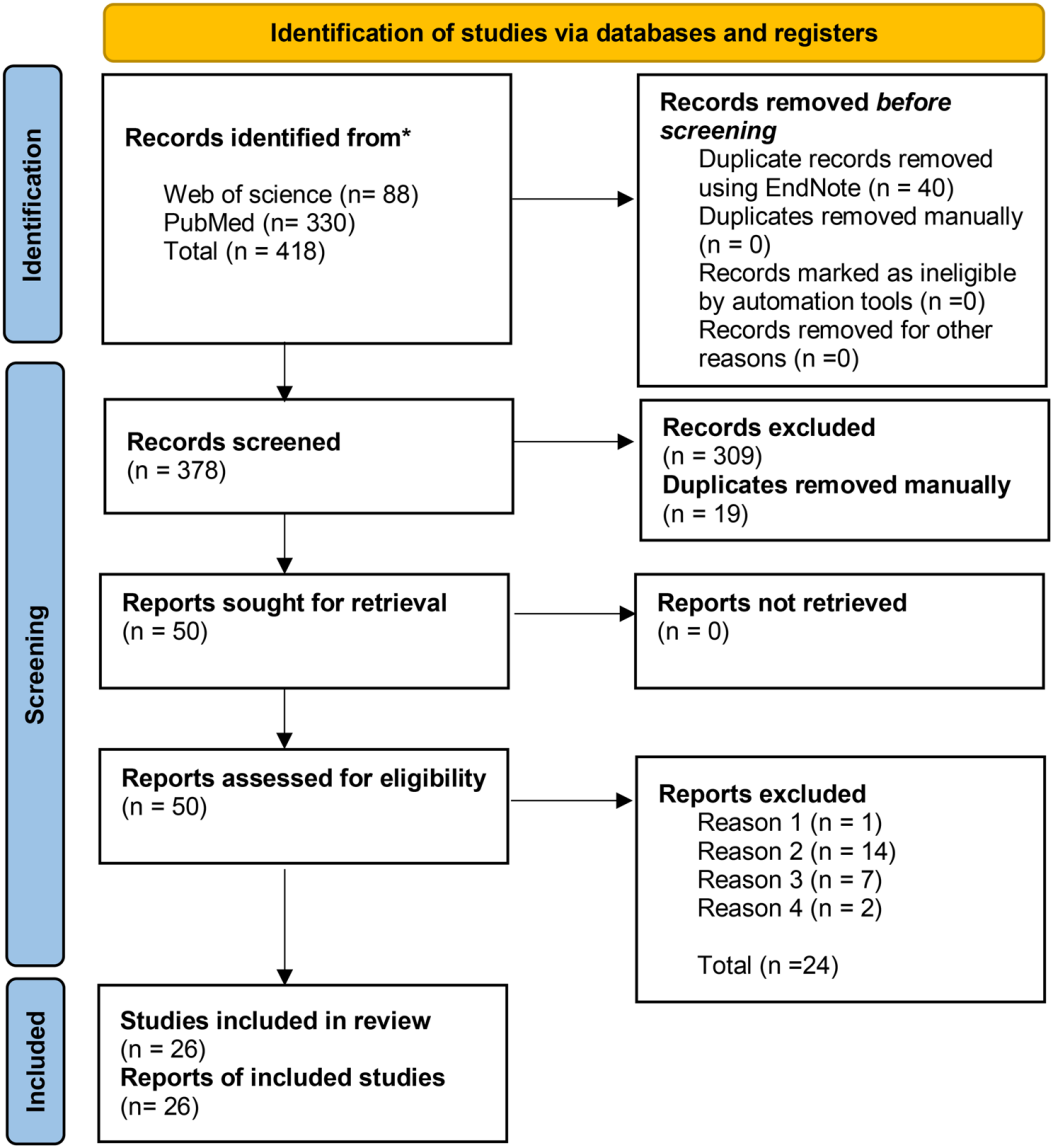
PRISMA flow diagram of the identification, screening, and selection process in numbers [18]. *Explanation of reasons for exclusion are presented in Table 4

A total of 378 articles were considered during the initial phase of screening based on their title and abstract. Identification, screening, and the inclusion process, are described in Fig. 1.

**Table 4:** Excluded articles and reason for exclusion (as shown in Table 5)

Authors	Title	Reason number	Exclusion reason
Myomin, T. Lim, S.	*Emergence and Development of Health Risk Communication Networks Among Street-Level Health Bureaucrats During the COVID-19 Pandemic Crisis in Myanmar*	2	Not mentioning community engagement strategies nor public health messaging.
Stadnick, N. A. Cain, K. Oswald, W. Watson, P. Nodora, J. Broyles, S. Lomeli, A. Escoto, A. Ibarra, M. Lagoc, R. Rabin, B.	*Insights from Immigrant and Refugee Communities Regarding COVID-19 Needs and Opportunities: A Mixed Methods Study*	1	Not from GMS.
Richard, Q. Alizon, S. Choisy, M. Sofonea, M. T. Djidjou-Demasse, R.	*Age-structured non-pharmaceutical interventions for optimal control of COVID-19 epidemic*	2	Not mentioning community engagement strategies nor public health messaging.
Suphanchaimat, R. Tuangratananon, T. Rajatanavin, N. Phaiyarom, M. Jaruwanno, W. Uansri, S.	*Prioritization of the Target Population for Coronavirus Disease 2019 (COVID-19) Vaccination Program in Thailand*	2	Not mentioning community engagement strategies nor public health messaging.
Phattharapornjaroen, P. Carlström, E. Sivarak, O. Tansuwannarat, P. Chalermdamrichai, P. Sittichanbuncha, Y. Kongtoranin, L. Phattranonuthai, R. Marlow, P. Winyuchonjaroen, W. Pongpasupa, N. Khorram-Manesh, A.	*Community-based response to the COVID-19 pandemic: case study of a home isolation centre using flexible surge capacity*	2	Not mentioning community engagement strategies nor public health messaging.
Ong, A. K. S. Prasetyo, Y. T. Yuduang, N. Nadlifatin, R. Persada, S. F. Robas, K. P. E. Chuenyindee, T. Buaphiban, T.	*Utilization of Random Forest Classifier and Artificial Neural Network for Predicting Factors Influencing the Perceived Usability of COVID-19 Contact Tracing “MorChana” in Thailand*	2	Not mentioning community engagement strategies nor public health messaging.
Sombultawee, K. Boon-itt, S. Bussanit, V.	*The Adoption of Protective Health Behaviors During the COVID-19 Pandemic in Thailand*	2	Not mentioning community engagement strategies nor public health messaging.
Truong, N. X. Ngoc, B. H. Ha, N. T.	*The Impacts of Media Exposure on COVID-19 Preventive Behaviors Among Vietnamese People: Evidence Using Expanded Protection Motivation Theory*	2	Not mentioning community engagement strategies nor public health messaging.
Tran, H. T. T. Lu, S. H. Tran, H. T. T. Nguyen, B. V.	*Social Media Insights During the COVID-19 Pandemic: Infodemiology Study Using Big Data*	2	Not mentioning community engagement strategies nor public health messaging.
Quach, H. L. Hoang, N. A.	*COVID-19 in Vietnam: A lesson of pre-preparation*	3	Does mention community engagement strategies or public health messaging, but too superficial.
Oo, M. M. Tun, N. A. Lin, X. Lucero-Prisno, D. E., 3rd	*COVID-19 in Myanmar: Spread*,* actions and opportunities for peace and stability*	3	Does mention community engagement strategies or public health messaging, but too superficial.
Chhim, S. Vong, W. I. Pa, K. Chhorn, C. Housen, T. Parry, A. E. Van Damme, W. Ir, P. Chhea, C.	*A descriptive assessment of the National Institute of Public Health’s contribution to the COVID-19 response in Cambodia*, *2020–2021*	3	Does mention community engagement strategies or public health messaging, but too superficial.
Leerapan, B. Kaewkamjornchai, P. Atun, R. Jalali, M. S.	*How systems respond to policies: intended and unintended consequences of COVID-19 lockdown policies in Thailand*	2	Not mentioning community engagement strategies nor public health messaging.
Van Nguyen, H. Van Hoang, M. Dao, A. T. M. Nguyen, H. L. Van Nguyen, T. Nguyen, P. T. Khuong, L. Q. Le, P. M. Gilmour, S.	*An adaptive model of health system organization and responses helped Vietnam to successfully halt the Covid-19 pandemic: What lessons can be learned from a resource-constrained country*	2	Not mentioning community engagement strategies nor public health messaging.
Tung, L. T. Thanh, P. T.	*Survey data on government risk communication and citizen compliance during the COVID-19 pandemic in Vietnam*	2	Not mentioning community engagement strategies nor public health messaging.
Yuduang, N. Ong, A. K. S. Prasetyo, Y. T. Chuenyindee, T. Kusonwattana, P. Limpasart, W. Sittiwatethanasiri, T. Gumasing, M. J. J. German, J. D. Nadlifatin, R.	*Factors Influencing the Perceived Effectiveness of COVID-19 Risk Assessment Mobile Application “MorChana” in Thailand: UTAUT2 Approach*	2	Not mentioning community engagement strategies nor public health messaging.
Van Minh, H.	*Proactive and Comprehensive Community Health Actions to Fight the COVID-19 Epidemic: Initial Lessons from Vietnam*	3	Does mention community engagement strategies or public health messaging, but too superficial.
Zweig, S. A. Zapf, A. J. Xu, H. Li, Q. Agarwal, S. Labrique, A. B. Peters, D. H.	*Impact of Public Health and Social Measures on the COVID-19 Pandemic in the United States and Other Countries: Descriptive Analysis*	3	Does mention community engagement strategies or public health messaging, but too superficial.
Chutiphimon, H. Thipsunate, A. Cherdchim, A. Boonyaphak, B. Vithayasirikul, P. Choothong, P. Vichathai, S. Ngamchaliew, P. Vichitkunakorn, P.	*Effectiveness of Innovation Media for Improving Physical Distancing Compliance during the COVID-19 Pandemic: A Quasi-Experiment in Thailand*	2	Not mentioning community engagement strategies nor public health messaging.
Dinh, L. Dinh, P. Nguyen, P. D. M. Nguyen, D. H. N. Hoang, T.	*Vietnam’s response to COVID-19: prompt and proactive actions*	3	Does mention community engagement strategies or public health messaging, but too superficial.
Duong, D. M. Le, V. T. Ha, B. T. T.	*Controlling the COVID-19 Pandemic in Vietnam: Lessons From a Limited Resource Country*	3	Does mention community engagement strategies or public health messaging, but too superficial.
Hinjoy, S. Tsukayama, R. Chuxnum, T. Masunglong, W. Sidet, C. Kleeblumjeak, P. Onsai, N. Iamsirithaworn, S.	*Self-assessment of the Thai Department of Disease Control’s communication for international response to COVID-19 in the early phase*	2	Not mentioning community engagement strategies nor public health messaging.
Briain, L. O.	*Jealous corona: Social media*,* musical propaganda and public health in Vietnam*	4	No full text available.
Thanh, P. T. Tung, L. T.	*Do government activities increase public compliance in the Covid-19 pandemic? Evidence from Vietnam*	4	No full text available.

We also conducted a screening calibration before reviewing all articles. The inclusion and exclusion criteria were used to determine the relevance of the title and abstract and whether to proceed with a full-text review [18]. We screened *n* = 30 articles initially to calibrate the screening scheme. After the calibration, 20% of the articles were screened and compared by both (*n* = 76). The lead author independently reviewed the remaining articles (*n* = 272), and those excluded were shared with the broader research team to ensure agreement on the excluded articles. In total, 50 articles were included for full-text review. In the second phase of the screening process, the included articles were read in full text to determine whether they met the inclusion and exclusion criteria. Then two reviewers independently conducted the study selection process in three stages: title and abstract screening, full-text review, and final inclusion. In the first stage, both reviewers screened titles and abstracts based on predefined eligibility criteria. Studies were included if they focused on public health messaging and community engagement during the COVID-19 pandemic in the GMS. Articles were excluded if they did not provide empirical findings, lacked relevance to RCCE, or focused solely on clinical or biomedical aspects of the pandemic response. In the second stage, the same two reviewers independently assessed the full texts of shortlisted articles for inclusion. Any discrepancies in study selection were discussed and resolved through consensus. If disagreements had persisted, a third reviewer was available to support the final decision.

#### Selection of articles

After the screening process, 26 articles were included, and 24 were excluded. Some articles mentioned public health measures implemented during the pandemic but were too superficial to be included in the review. ‘Superficial’ refers to those articles that were published in 2020 to learn from measures made in the early phase of the pandemic but the information mentioned did not detail the relevant initiatives, so were excluded. Table 5 provides an overview of reasons for exclusion and the number of articles in each category.

**Table 5:** Reasons for exclusion after full-text reading and the total amount of articles in each category

#	Reason for exclusion	Total	Percentage (%)
1	Not from Greater Mekong Subregion.	1	4,17
2	Not mentioning community engagement strategies nor public health messaging.	14	58,33
3	Does mention community engagement strategies or public health messaging, but too superficial.	7	29,17
4	No full text available.	2	8,33
	Total	24	100

### Ethics

Ethical review was not required for this rapid review.

## Results

We describe the study characteristics, and then the content and a narrative description of the topics in the articles. The narrative description is structured into two subsections on community engagement strategies and public health messaging initiatives.

### Study characteristics

In this section, each source of evidence is presented with its characteristics according to the PRISMA checklist [19]. Table 6 presents the title, author(s), year, methodology, country, method and aim and themes of included articles. Table 7 shows the countries in which the initiatives presented in publications were carried out, and the years of publication. None of the articles included were published in 2024, so 2024 is removed from the table. The same process was applied to the regions in China (Yunnan Province and Guangxi) and the country of Laos, as none of the included articles were from these areas. Most articles were published with results from Vietnam (*n* = 14 + 2) and Thailand (*n* = 9 + 2), and the countries with the fewest publications were Cambodia (*n* = 1) and Myanmar (multinational) (*n* = 1). There were few articles in 2020 (*n* = 2), and the peak was in 2021 with 13 publications.

**Table 6:** Presentation of included articles title, author, year, methodology, country, method, aim and themes

#	Title and year	Author	Country	Method	Purpose of the study (aim)	Themes and sub-themes
1	*Feasibility of Intersectoral Collaboration in Epidemic Preparedness and Response at Grassroots Levels in the Threat of COVID-19 Pandemic in Vietnam* 2020	Le, H. T. Mai, H. T. Pham, H. Q. Nguyen, C. T. Vu, G. T. Phung, D. T. Nghiem, S. H. Tran, B. X. Latkin, C. A. Ho, C. S. H. Ho, R. C. M.	Vietnam	Quantitative	To evaluate the collaborative mechanism between health and community service workers with intersectional organizations at grassroots levels in Vietnam.	Pandemic preparedness, community networks, inter-/multisectoral collaboration
2	*Responding to the COVID-19 s wave in Thailand by diversifying and adapting lessons from the first wave* 2021	Rajatanavin, N. Tuangratananon, T. Suphanchaimat, R. Tangcharoensathien, V.	Thailand	Descriptive	To analyze how the response to the first wave informed the informed the second wave response and identifies lessons for further improvement.	Active case finding, targeted strategy, inter-/multisectoral collaboration
3	*When communist propaganda meets western public relations: Examining Vietnam’s government pandemic communication* 2023	Le, T. L. Block, E.	Vietnam	Literature review	To explore the communication strategies used by Vietnam’s communist government during the earlier phases of the COVID-19 pandemic.	Risk communication strategies, pandemic communication framework
4	*News frames for COVID-19. A comparison of Australian (Australian Broadcasting Corporation) and Vietnamese (Tuoi Tre Online) online news services in two critical weeks in 2020* 2023	Le, V. T. Green, L.	Vietnam	Literature review	To investigates the differences and similarities between the news frames used by online mainstream media in Vietnam and Australia when reporting COVID-19 in the early waves of the pandemic.	Risk communication strategies, Framing of pandemic
5	*Risk Communication Distributed among Migrant Workers during the COVID-19 Crisis in Thailand: Analysis on Structural and Networking Gaps* *2022*	Kosiyaporn, H. Julchoo, S. Papwijitsil, R. Uansri, S. Phaiyarom, M. Sinam, P. Suphanchaimat, R.	Thailand	Mixed method	To explore health risk communication structure and processes and identify the communication network of migrant workers during the COVID-19 pandemic in Thailand	Risk communication strategies, inter-/multisectoral collaboration
6	*“Having vaccines is good but not enough”: Requirements for optimal COVID-19 immunization program in Vietnam* 2023	Doan, L. P. Dao, N. G. Nguyen, D. C. Dang, T. H. T. Vu, G. T. Nguyen, L. H. Vu, L. G. Le, H. T. Latkin, C. A. Ho, C. S. H. Ho, R. C. M.	Vietnam	Descriptive	To share information about Vietnams vaccine strategy.	Risk communication strategies, organization of vaccination, human resources
7	*Community responses to COVID-19 pandemic first wave containment measures: a multinational study* 2021	Aung, M. N. Stein, C. Chen, W. T. Garg, V. Saraswati Sitepu, M. Thu, N. T. D. Gundran, C. P. D. Hassan, M. R. Suthutvoravut, U. Soe, A. N. Nour, M. Gyi, K. K. Brandl, R. Yuasa, M.	Vietnam, Myanmar and Thailand	Qualitative	To explore community responses to COVID-19 containment measures in different countries and synthesize a community response model.	Risk communication strategies, previous pandemic experience, health literacy
8	*Effects of public health interventions on the epidemiological spread during the first wave of the COVID-19 outbreak in Thailand* 2021	Triukose, S. Nitinawarat, S. Satian, P. Somboonsavatdee, A. Chotikarn, P. Thammasanya, T. Wanlapakorn, N. Sudhinaraset, N. Boonyamalik, P. Kakhong, B. Poovorawan, Y.	Thailand	Literature review	To report about Thailand’s public health interventions and gather lessons learned from the first wave.	Public health interventions, community health infrastructure, compliance
9	*COVID-19: Lessons from Thailand* 2021	Srisawat, N. Iamsirithaworn, S. Tantawichiein, T. Thisyakorn, U.	Thailand	Descriptive	To discuss Thailand’s strategy for containing the disease, the management of severe COVID-19 patients, and future perspectives on COVID-19.	Multi-layered public health response, primary healthcare infrastructure, pandemic preparedness
10	*The COVID-19 containment in Vietnam: What are we doing?* 2020	Huynh, T. L. D.	Vietnam	Descriptive	To share information about Vietnam’s response to COVID-19.	Preventive measures, risk communication strategies, inter-/multisectoral collaboration
11	*From disease- to people-centred pandemic management: organized communities*,* community-oriented primary care and health information systems* 2023	Leyns, C. Willems, S. Powell, R. A. Camacho, V. Fabrega, R. De Maeseneer, J. Rawaf, S. Mangtani, P. El-Osta, A.	Thailand	Literature review	To analyze whether and how people’s central role can promote a successful pandemic response.	Community networks, pandemic preparedness, human resources
12	*In the interest of public safety: rapid response to the COVID-19 epidemic in Vietnam* 2021	Nguyen, T. V. Tran, Q. D. Phan, L. T. Vu, L. N. Truong, D. T. T. Truong, H. C. Le, T. N. Vien, L. D. K. Nguyen, T. V. Luong, Q. C. Pham, Q. D.	Vietnam	Descriptive	To summarize the status of the COVID-19 epidemic in Vietnam, highlight key response successes, examine factors that prompted the implementation of specific public health actions, and describe the impact of these actions.	Pandemic preparedness, inter-/multisectoral collaboration, communication of health promotion measures
13	*COVID-19 initial preparedness and response in Vietnam during the first six months of the pandemic and the lessons for Sendai framework implementation* 2021	Linh, T. N. Q. Tran, T. Y. H. Shaw, R.	Vietnam	Literature review	To synthesize information about Vietnam’s pandemic response and discuss how the Sendai framework can be implemented to strengthen pandemic response capacity.	Pandemic preparedness, timely policies’ implementation, risk communication strategies
14	*Governance and state-society relations in Vietnam during the COVID-19 pandemic* 2022	Taniguchi, M.	Vietnam	Descriptive	To describe how the Vietnamese government attempted to control COVID-19 and how citizens responded to state policies.	Risk communication strategies, community networks
15	*Community engagement in the prevention and control of COVID-19: Insights from Vietnam* 2021	Ha, B. T. T. Ngoc Quang, L. Quoc Thanh, P. Duc, D. M. Mirzoev, T. Bui, T. M. A.	Vietnam	Qualitative	To describe new knowledge on effective approaches to ensure effective responses to public health measures like lockdowns, travel restrictions and social distancing. Highlighting experiences of CE in Vietnam.	inter-/multisectoral collaboration, capacity strengthening, risk communication strategies
16	*The Contribution of Digital Health in the Response to Covid-19 in Vietnam* 2021	Bui, L. V. Ha, S. T. Nguyen, H. N. Nguyen, T. T. Nguyen, T. P. Tran, K. Tran, T. V. Nguyen, T. H. Tran, T. H. Pham, N. D. Bui, H. M.	Vietnam	Literature review	To provide an overview on implementing digital health technologies during COVID-19 in Vietnam.	Digital health, risk communication strategies
17	*Community surveillance of COVID-19 by village health volunteers*,* Thailand* 2021	Kaweenuttayanon, N. Pattanarattanamolee, R. Sorncha, N. Nakahara, S.	Thailand	Descriptive	To describe the unique system of health volunteers in Thailand.	Human resources
18	*COVID-19 public health and social measures: a comprehensive picture of six Asian countries* 2022	Foo, C. Verma, M. Tan, S. M. Haldane, V. Reyes, K. A. Garcia, F. Canila, C. Orano, J. Ballesteros, A. J. Marthias, T. Mahendradhata, Y. Tuangratananon, T. Rajatanavin, N. Poungkantha, W. Mai Oanh, T. The Due, O. Asgari-Jirhandeh, N. Tangcharoensathien, V. Legido-Quigley, H.	Thailand and Vietnam	Literature review	To analyze public health and social measures implemented in six Asian countries through an in-depth case study analysis.	Health system capacity, community engagement
19	*An examination of Thailand’s healthcare system and strategies during the management of the COVID-19 pandemic* 2021	Issac, A. Radhakrishnan, R. V. Vijay, V. R. Stephen, S. Krishnan, N. Jacob, J. Jose, S. Azhar, S. M. Nair, A. S.	Thailand	Descriptive	To examine the strategies behind the success of Thailand in containing the COVID-19 pandemic.	Pandemic preparedness, community networks, risk communication strategies
20	*The heterogeneity of the COVID-19 pandemic and national responses: an explanatory mixed-methods study* 2021	Chen, Y. Y. Assefa, Y.	Thailand	Literature review	To understand the strengths of low-burden countries and weaknesses of hotspot countries’ responses to COVID-19.	Risk communication strategies, community engagement, inter-/multisectoral collaboration, public health capacity
21	*Descriptive assessment of COVID-19 responses and lessons learnt in Cambodia*,* January 2020 to June 2022* 2023	Chhim, S. Ku, G. Mao, S. Put, W. V. Van Damme, W. Ir, P. Chhea, C. Or, V.	Cambodia	Descriptive	To describe the epidemiological phases, response phases, strategy and lessons learnt in Cambodia between2020 2022.	Early response system, healthcare capacity strengthening, cooperation with the public
22	*The role of government risk communication in public health emergencies: evidence from the COVID-19 pandemic* 2022	Thanh, P. T. Tung, L. T.	Vietnam	Quantitative	To investigate the role of risk communication in shaping the public’s knowledge, risk perception, and compliance with safety measures during COVID-19.	Risk communication strategies, compliance with safety measures
23	*Can risk communication in mass media improve compliance behavior in the COVID-19 pandemic? Evidence from Vietnam* 2022	Thanh, P. T. Tung, L. T.	Vietnam	Quantitative	To investigate the relationship between risk communication in mass media and the relation to public risk perception, understanding, and compliance	Risk communication strategies, compliance with safety measures, risk perception
24	*No one left behind: risk communication to the street vendors during COVID-19 social distancing* 2022	Thanh, P. T. Nguyen, H. T. H. Ngan, L. B. Nguyen, D. M. D. Phan, G. H. Nguyen, T. M. N.	Vietnam	Quantitative	To examine the roles of risk communication work in enhancing COVID-19 risk perception and adoption of COVID-19 preventive behaviors among street vendors.	Risk communication strategies, risk perception, preventive behaviors
25	*Public health policies and health-care workers’ response to the COVID-19 pandemic*,* Thailand* 2021	Nittayasoot, N. Suphanchaimat, R. Namwat, C. Dejburum, P. Tangcharoensathien, V.	Thailand	Descriptive	To review of the government policies that enabled early containment and enhanced health-care workers’ capacity to respond effectively to the pandemic.	capacity of health-care workers, occupational safety, public health policies
26	*COVID-19 control in Vietnam* 2021	Van Tan, L.	Vietnam	Descriptive	To reflect on how Vietnam has succeeded in controlling the virus.	Pandemic preparedness, compliance with safety measures

**Table 7:** Publications by countries and years, including publications referring to more than one country

	Years of publication					
**Countries of initiatives**	2020	2021	2022	2023	Total	Percentage per country (%)
Cambodia				1	1	3,85
Thailand		7	1	1	9	34,62
Vietnam	2	5	4	3	14	53,85
Vietnam and Thailand			1		1	3,85
Vietnam, Thailand, Myanmar		1			1	3,85
Total	2	13	6	5	26	100
Percentage per year (%)	7,69	50	23,08	19,23	100	-

#### Type of included articles

Some of the articles included several methodologies for data collection but are only described under the method category relevant to the research question in this paper. One article in this section is presented twice, as it uses a study design combining quantitative and qualitative data collection [20]. The qualitative and quantitative data from this study were distinct and relevant to the results and are therefore included in each category.

##### Qualitative data

Three studies use qualitative methods to investigate aspects of community engagement and communication strategies in response to the COVID-19 pandemic. Ha et al. [21] and Kosiyaporn et al. [20] employed in-depth interviews to explore community engagement approaches and health risk communication structures, focusing on Vietnam and Thailand, respectively. Aung et al. [22] utilized an open-ended questionnaire across multiple countries to understand community responses to infection control measures and social distancing, culminating in a model illustrating underlying factors influencing these responses.

##### Quantitative data

We included five quantitative studies (one of which applies a mixed-methods approach) on different aspects of public health messaging and community engagement during the COVID-19 pandemic. Le et al. [23] surveyed 581 respondents on the efficiency of involving community service workers in healthcare activities. Thanh authored three articles: one surveyed street vendors in Vietnam (*n* = 105) [24], while two others surveyed citizens in Vietnam during the pandemic (*n* = 467 each) [25]. These studies focused on risk communication, public understanding, and compliance with health measures [25, 26]. Kosiyaporn et al. [20] conducted a social network analysis on migrant workers in Thailand (*n* = 56) to understand their communication network during the pandemic, involving various stakeholders like local governments and NGOs.

##### Literature review

Several literature reviews analyzed Thailand and Vietnam’s responses to the COVID-19 pandemic. Two of the articles focused on Thailand; Triukose et al. [27] reported on Thailand’s public health interventions, reviewing Thai Government documents and information from the Centre for COVID-19 Situation Administration (CCSA) to collect information on policies and public health measures taken during COVID-19. Chen and Assefa [28], examined the strengths of low-burden countries and weaknesses of hotspot countries’ responses to COVID-19 through a review of COVID-19 literature. Bui et al. [29] reviewed the Vietnamese adoption of digital health technologies, by reviewing official documents on the topic and one Google search focusing on digital health and COVID-19. Linh et al. [30] analyzed the pandemic response in Vietnam by synthesizing the literature on response measures to the COVID-19 pandemic and comparing these actions with the Sendai Framework. The aim of the study was to discuss how the framework could be implemented to strengthen pandemic response capacity. Le and Block [31] and Le and Green [32] analyzed media content. Le and Block [31] analyzed government and news media, as well as social media texts, to identify strategies that were dominant during COVID-19. Le and Green [32] identify similarities and differences between Vietnamese and Australian news media during the pandemic. Two of the articles analyzed information from several countries, with one looking at public health measures implemented in six Asian countries [33], and the other looking at how people-centred pandemic management could promote successful pandemic response [34].

##### Descriptive

We identified eleven articles as descriptive. Doan et al. [35] and Huynh [36] wrote about Vietnam’s vaccine strategy and the government’s public health policies to contain the COVID-19 pandemic. Huynh’s article describes information sharing on the measures taken in the early phase of COVID-19, including information about the Vietnamese government’s communication strategy. Doan et al. [35] provide a short article with information about a communication plan developed to mobilize society to participate in vaccination. However, they do not state how the secondary data was selected for the article. In the articles from Vietnam, Taniguchi [37] and Le and Block [31] investigated how an authoritarian management style, like that of the Vietnamese government, attempted to control COVID-19 and how citizens responded to state policies. The later study used secondary data. The second descriptive article from Vietnam aimed to provide a summary of the status and responses to the COVID-19 epidemic by examining factors that prompted the implementation of certain public health actions and describing the impact of these actions [38]. The method was not mentioned in the article. A descriptive article from Cambodia describes the epidemiological phases, response phases, strategies, and lessons learned in Cambodia between 2020 and 2022, using data from the Ministry of Health about confirmed cases of COVID-19, deaths, and measures taken to prevent the spreading of the pandemic [39].

Two articles examine Thailand’s early pandemic response, focusing on actions taken and policies implemented to curb the spread of COVID-19. Rajatanavin et al. [40] analyzed how Thailand’s response to the first wave shaped strategies for the second wave. Their study relied on secondary data, including official reports, surveys, secondary data analysis, and minutes from the Public Health Emergency Operation Centre. Another reviewed government policies aimed at achieving early containment and supporting healthcare workers in managing the crisis [41]. However, their article relies on secondary sources without clarifying the selection criteria. Other work describes how Thailand responded to the COVID-19 pandemic [42, 43] including the strategies implemented and information about the approach of village health volunteers (VHV). Some used secondary data from sources such as the newspaper The Bangkok Post, press releases from the Thai government and information from the WHO [44].

### Synthesis of included articles

We structure the results across the two broad themes of public health messaging and community engagement initiatives. The subthemes include government communication strategies, communication channels, engagement communication, response to misinformation, reaching vulnerable groups, community engagement and mobilization, incentives for participation in public health measures, multisectoral collaboration and engaging with vulnerable groups.

#### Public health messaging initiatives

##### Government communication strategies

Centralized and regularly updated COVID-19 information was sent from the governments of Thailand, Cambodia, and Vietnam as a part of the pandemic response communication strategies [27–29, 32, 36, 38, 39, 41, 43–45]. Nittayasoot et al. [41] describe how the daily briefs provided by the government in Thailand contained epidemiological updates on the regional, national, and global situation and the preventive measures citizens were required to adopt. In Vietnam, a comprehensive strategy for risk communication was published in January 2020, and this was used during the pandemic response to COVID-19 [38, 45]. The strategy included a section on communication during the public health crisis and focused on timeliness, transparency, precision, and reliability of COVID-19 information to gain community involvement [38]. Both Vietnam and Thailand developed COVID-19 webpages, where the official information to the public was published [27, 29, 37]. Thailand established the Center for COVID-19 Situation Administration (CCSA), who were used as a single command center to ensure coherent communication about the situation [27, 40, 43]. A similar approach was applied in Vietnam, where daily reports, including public health information and scientific evidence of the pandemic, were published on the official portal on COVID-19, which is a webpage administered by the Ministry of Health in Vietnam [29, 31]. In Cambodia, the Ministry of Health shared official information with the country on a regular basis, through various Ministry of Health communication channels [39]. The government of Vietnam applied a *news management practice* during the pandemic, which is a practice where the government sets the agenda of the media [31]. Media houses in Vietnam were asked to use the COVID-19 portal as the source of communication about COVID-19, ensuring streamlined and coherent information flow to the public [31]. The Vietnamese media were closely monitored by the government and directed to share and increase the coverage of COVID-19 information, resulting in 560,000 news articles about COVID-19 from the 1st of February to the 31st of May 2020 [31]. Le and Green [32] identified the way of communication as a one-way and top-down communication, using an “information delivery model.” It is argued that the information shared was from the biomedical authorities, seeing the recipients of the information as passive, and simply receiving knowledge from above. Le and Block [31] argue that the communication style was a hybridized traditional monologic propaganda, with public relations (PR) dialogic communication strategies where the government set the media agenda to combat alternative dialogues, and influence people’s understanding of the COVID-19 pandemic.

##### Communication channels

Thailand, Cambodia, and Vietnam used different communication channels to disseminate public health information about COVID-19, aiming to reach different groups in the population [20, 30, 39, 41]. The information from the centralized authorities in both Thailand and Vietnam was conveyed via TV, radio, online infographics, and videos [20, 26]. Findings from Vietnam showed that different parts of the population use and require different means of communication. Young people receive risk communication through social media, and the elderly receive it through more traditional media such as TV and radio [26].

During the pandemic response in Vietnam, Thailand, and Cambodia, social media emerged as a crucial tool for disseminating information. In Vietnam, official authorities utilized social media like Facebook, leveraging its vast user base to reach a significant portion of the population [30, 37]. In both Cambodia and Thailand, social media were regularly used to share information and communicate with the population about COVID-19 [28, 39].

Moreover, mobile applications were developed to monitor the spread of COVID-19 and provide updates on pandemic developments and health precautions. In Vietnam, the government introduced two applications: one for contact tracing and another for health declaration [30, 37]. Notably, the contact-tracing application was downloaded by 14.9 million users, constituting 14% of the Vietnamese population [37]. In Thailand, an application was developed to monitor the spread of disease, and this application was used by the population or with the help of VHV [42]. In addition to mobile apps, phone calls and SMS were used to provide information about COVID-19. Vietnamese authorities implemented a short prevention message with every smartphone call, ensuring widespread awareness [30, 37]. Furthermore, the government sent 6 billion SMS containing public health messaging to Vietnamese citizens [30, 36].

In Vietnam, the government also collaborated with Vietnam National Television (VNT) to broadcast health literacy content and updates on the pandemic response [31]. Weekly press conferences were held and sent on VNT TV channels to inform the public about government initiatives [31]. The same applies to Thailand, where the authorities used mass media for risk communication [20]. Furthermore, in Vietnam, the government used traditional communication methods such as loudspeakers to deliver information to the public [37]. Loudspeakers were used as a communication tool during the war in Vietnam in the 1960–1970 s and are considered to have recalled images of the US invasion during the war, especially when the means of communication was combined with war metaphors, uniting people against the new “enemy” (COVID-19) [31].

##### Engagement communication

The governments of Vietnam, Cambodia, and Thailand were all using specific discourse or communication strategies to engage with the population in their country, depending on the country’s history and context [31, 39]. Vietnam communications used the war in Vietnam to motivate people to engage in the pandemic response, using phrases such as “Every citizen is a soldier fighting the disease [32, 37]. According to Le and Block [31], the Vietnamese government used propaganda slogans to communicate, appealing to emotions like fear and love of other countrymen and further appealing to the culture of collectivism, patriotism, and solidarity. In the pandemic’s Delta wave of COVID-19, the Vietnamese government shifted the message to focus on “living with the virus” [31]. The Government of Cambodia called upon the population’s engagement using solidarity to motivate them to follow the required pandemic rules and regulations [39]. Meanwhile, another discourse was applied in Thailand as they introduced “Big Cleaning Week” to create awareness of COVID-19 and personal hygiene in the workplace, at home, and in public spaces [43].

Many of the governments included here have used different creative initiatives to reach the population with public health messages, like songs, poems, paintings, short messages, videos, and infographics [25, 30, 31, 37, 39]. Vietnam used creative ways of communication and labelled music as “the weapon to fight COVID-19” [31]. This approach resulted in the health communication campaign “Ghen Co Vy” (“jealous coronavirus”), an adaptation of a pop song from 2017, with a text about the virus and measures to be taken to control and prevent the virus from spreading [30, 37]. Through the song’s text and animation, the Vietnamese Ministry of Health used the song to call upon the Vietnamese collective culture and the citizens’ individual responsibility [31]. The song went viral on social media, with over 70 million views in March 2021 [45], and the total number of views in April 2024 was 121 million [46].

Furthermore, the Vietnamese government used catchy slogans to communicate health messages and motivate people to follow guidelines and regulations [30]. An example is the hashtag used on TikTok, #Onhavanvui which translates as #Stayinghomeisfun, that gained more than eight million views during July 2020 [37]. Other examples include # Staying at home is patriotic, # Put on a face mask, wash hands often, and # The virus is your enemy [30]. To share the messages, the Vietnamese government used celebrities, for instance, by sharing slogans like # Stay strong on social media [37]. The Cambodian Government also used simplified and short messages to inform the public. An example was the “3 DOs and 3 DON’Ts”, including ‘do’ handwashing and ‘don’t’ go to crowded places [39].

##### Response to misinformation

Myanmar, Vietnam, Cambodia, and Thailand have all incorporated misinformation management into their communication strategies. Notably, Myanmar, Vietnam, and Thailand have established surveillance teams dedicated to tackling rumours and false information circulating on social media platforms concerning COVID-19 [22, 37, 43]. In Thailand, the Centre for COVID-19 Situation Administration (CCSA) had the responsibility of identifying and countering misinformation related to COVID-19. Misinformation was tracked down immediately and addressed in the news in Thailand [28]. Similarly, in Myanmar, surveillance teams were formed to combat COVID-19-related rumours and false information on platforms like Facebook [22]. In Cambodia, the Ministry of Health regularly disseminated information to counteract myths, false information, and rumours on social media [39]. Vietnam adopted a stringent approach, actively tracking and penalizing those responsible for spreading misinformation, often imposing fines, or other strict penalties [32, 38].

##### Reaching vulnerable groups

Migrant workers struggle to access important information about public health threats because of many barriers, including language barriers [20, 40]. In Vietnam, information about COVID-19 was shared in several languages [28]. The government communicated in English, Chinese, French, and Korean to reach migrants, minority ethnic groups, and tourists in the country with information about COVID-19 [31, 37, 38]. The translated information was disseminated through traditional media, social media, and loudspeakers [31]. In Thailand, they established a network of Migrant Health Volunteers (MHV) to reach migrants with information about COVID-19. The migrants then received information from people who spoke the same language [40]. The MHV was identified as a key source of information for most migrant communities [20]. Thanh et al. [24] found that street vendors used several means of communication to receive information about COVID-19. 97% of the street vendors received information from friends and relativesand the most used communication channels were TV at 65%, and social media at 62%. 94% had received information from at least three channels [24].

#### Community engagement initiatives

##### Community engagement and mobilization

Two articles highlight utilizing pre-existing community networks and structures for effective pandemic response and community engagement. Vietnam and Thailand’s governments used existing structures for information sharing and contributions to pandemic response efforts [23, 27]. In Thailand, the VHVs were utilized as a direct channel for health authorities to provide information and execute pandemic prevention and control activities. With over a million volunteers nationwide [44] and 15,000 public health volunteers in Bangkok, VHVs reached over 11 million households during the pandemic [27]. Health volunteers are persons in the community working with follow-up on health-related tasks, especially in rural areas [27]. Since 2009, each volunteer has received a monthly honorarium for their work [34]. Similarly, in Vietnam, findings show that the Vietnamese government systematically expanded youth networks from local to national levels, strengthening existing structures to control the pandemic [23].

Mobilizing local actors to share information about the pandemic was described as essential to gaining the trust of the population. It contributes to community engagement and enables people to follow guidelines and regulations in response to the pandemic [23, 27]. In Thailand research describes how the VHVs gained trust in the community because they belong to the community [27]. They were used as collaboration partners between local and national levels, and could access people who were hard to reach, such as the elderly, people living in poverty, and people with disabilities or illness [27]. The authors argue that the role of the VHV in Thailand was the key factor in helping control COVID-19, as they accessed and reached the communities with important information and activities for prevention and control.

Furthermore, the VHVs conducted surveillance and monitoring of the health of those in their community [27, 40, 42–44]. After the lockdown in Thailand in March 2020, many workers went home from central to rural areas [42]. Srisawat et al., [44] describe how the VHVs were used to identify returnees from the cities to rural areas, monitor their health, and register if the returnees showed any signs of symptoms of COVID-19. The VHVs visited around 14 million households in March and April 2020 [42]. During this time, they monitored 809,911 returnees and provided them with information on mandatory quarantine for 14 days [42]. The VHVs requested the use of a mobile application for registering symptoms, and in cases where individuals could not use the application, the VHVs monitored their health daily [42]. Vietnam also used local actors to conduct activities in the pandemic response [23, 33, 37]. Medical students in Vietnam were enrolled locally to assist the community with several activities such as epidemiological investigations, quarantine guidance, and blood sampling [23]. The results from the article by Le et al., [23] report that medical students involving community service workers in health-related activities at a grassroots level perceived more efficiency than other health workers [23]. There were also established community COVID-19 supervision groups containing VHVs, youth volunteers, or volunteers from the women’s union, and these groups were responsible for sharing health information and promoting compliance with implemented public health social measures [33]. Taniguchi [37] also describes the COVID-19 supervision groups. However, the groups mentioned in the article by Taniguchi [37] also contain members of the police or military. The supervision groups followed up on state directives at a local level [37]. Another local initiative described by Taniguchi [37] was the “propaganda groups,” consisting of VCP (Vietnamese Communist Party) members, which covered forty to fifty households, conducting activities such as door-to-door checks to ensure people complied with the pandemic prevention and control measures [37]. The VCP communicators consisted of 20,000 people, and they disseminated information from a central level down to the local community [37].

##### Incentives for participation in public health measures

Several strategies have been used to motivate populations to follow public health measures . In Thailand it was possible to win a cow if you got vaccinated [33] and VHVs got a 50% increase in their monthly salary during COVID-19 [34, 41]. On the other hand, while collaboration played a crucial role in mitigating the spread of COVID-19, enforcement measures were also implemented to ensure compliance with government regulations. For instance, in Vietnam, fines were given to individuals who did not follow the mask-wearing rules in public spaces [38]. Similarly, in Thailand, the VHVs were responsible for monitoring individuals required to undergo quarantine, and those found violating the requirements were reported and could face arrest [43].

##### Multisectoral collaboration

Multisectoral collaboration was cited frequently as a part of the strategy to engage the whole society in the fight against COVID-19 [21, 34, 36, 37, 44]. Ha et al. [21] describe how Vietnam’s community engagement approach used multi-sectional collaboration as one main factor in their strategies. This can be seen where structures to combat COVID-19, such as COVID-19 committees, engaging migrant workers, and capacity building were established [21]. The afore mentioned COVID-19 supervision groups from Vietnam, comprised of workers and volunteers from the community, exemplify the collaborative effort from various segments of society. Similarly, Thailand used a whole-of-society approach, emphasizing collaboration among all sectors in the country as a strategy for containing COVID-19 [34, 44]. This collaboration between citizens, the private sector, and civil society aimed to reduce the impact on vulnerable populations [34].

##### Engaging vulnerable groups

The response to the second wave of COVID-19 in Thailand was considered to be unsuccessful [20, 40, 44]. Migrant community members found it difficult to engage with the information shared, and this hindered their ability to follow public health and social measures as part of a pandemic response [40]. Additional barriers were limited testing due to a fear of deportation or legal actions, language barriers, limited access to timely information, and low literacy [20, 40]. However, MHVs played a significant role in supporting contract tracing and capacity building, as they knew the community and spoke the same dialect as many workers [40].

## Discussion

This rapid review examined public health messaging and community engagement initiatives in the GMS during the COVID-19 pandemic. The findings describe diverse, novel and traditional approaches to communication and engagement, that provide insights into the strengths and challenges of risk communication and community engagement (RCCE) strategies in the region. We discuss first where our findings relate to existing literature broadly, then discuss engagement, dialogue and ethics

Our findings resonate with studies from other regions where different RCCE structures — especially top-down versus community-led models — influence both compliance and trust. For instance, a study in Malawi showed that community-mobilization strategies (dialogue, peer education, and social media) were more accessible and trusted by populations in rural areas than purely mass media or government announcements, particularly when implementers blended multiple communication channels to respond to local concerns [47]. A scoping review found that combining national policies with locally adapted implementation (including local leaders and cultural tailoring) produced more effective outcomes, whereas rigid, purely centralized approaches often failed to resonate with marginalized or geographically isolated communities [48]. Similarly to our findings from Thailand, in 13 African countries, RCCE strategies that included active community engagement (such as dialogue meetings, peer educators, local health workers) showed better uptake of prevention behaviours and vaccine acceptance than strategies relying mainly on top-down messaging [9]. A systematic review further identifies that trust, inclusion, feedback loops, and recognizing local structures act as major determinants of communication effectiveness particularly when RCCE is community-centred rather than imposed from above [49].

### Engaging through communication

We see that centralized communication systems are pivotal for coordinating and aligning public health messaging, which is consistent with WHO recommendations for risk communication systems to ensure coherence and prevent public confusion [4]. Vietnam exemplified this, maintaining consistent messaging throughout the pandemic [32]. In contrast, European countries such as Germany and Sweden are cited as constantly changing messaging and the UK saw over 60 changes to COVID-19 guidelines within a year, causing public confusion and undermining compliance [50]. The GMS governments generally took a leading role in communicating risks to their populations, avoiding the misinformation and myths that hindered responses in other regions like Tanzania [9]. WhileVietnam’s highly centralized communication model had notable strengths, particularly in ensuring message consistency and public compliance, the effectiveness must be weighed against alternative approaches. Decentralized models, as seen in Germany and South Korea, allowed for more tailored, region-specific messaging, increasing public engagement and responsiveness to local concerns [50]. While Vietnam’s approach prevented contradictory messaging, it also limited opportunities for community-level adaptations, potentially reducing the effectiveness of communication among diverse populations. Emotive, tailored discourse was also deployed alongside the centralised approaches. Vietnam’s use of war metaphors invoked resilience, framing the virus as a common enemy to be defeated collectively. Cambodia adopted a solidarity-driven discourse, paralleling strategies in European countries like Germany, where messages of patience and unity promoted compliance [50]. While social media proved effective in raising awareness, its role in shaping public understanding of risks was limited. Findings from Vietnam indicate that mass media exposure was more strongly associated with compliance than social media use [20]. This raises concerns about whether risk communication merely elicits obedience rather than enabling informed decision-making [4]. However, a study from Portugal and Spain has emphasized the use of digital technology and communication for the elderly as a tool for limiting public confusion and effective communication [51]. Ensuring elderly groups have sufficient education in digital technologies can facilitate trusted information sharing between them and their younger relatives [51]. Using more accessible digital applications may help rebuild trust in information sharing in contexts where messaging was sporadic and changing [51].

### Dialogue and two-way communication

Dialogue is considered an essential component of effective RCCE, as it allows authorities to understand community concerns, address misinformation, and adapt interventions to local realities [38]. Yet, across the GMS, evidence of genuine two-way communication remains limited. Vietnam’s strategy, highly centralized and consistent, largely relied on one-way, top-down messaging. This raises questions about whether community perspectives were meaningfully incorporated into the response [39]. While live-streaming initiatives were introduced as interactive platforms, media analyses suggest these were more symbolic than substantive, with little evidence that public concerns were represented or acted upon [24]. Moreover, the suppression of dissent — including the arrest of individuals critical of government policies — further restricted the space for open dialogue, suggesting that public engagement was tolerated only insofar as it reinforced official narratives [24]. This dynamic contrasts with participatory communication models observed in other contexts. In New Zealand, for example, government leaders actively engaged with citizens through open forums and press briefings that allowed real-time questioning, providing transparency and accountability [41]. Communication techniques such as this are a clear governance choice, that either reinforces or undermines the legitimacy of public health measures. Within the GMS, Thailand’s VHVs provided a notable exception by facilitating two-way communication at the community level. Their door-to-door visits enabled both dissemination of public health information and collection of community feedback [35]. Similar models have proven successful elsewhere, including Ghana, where community volunteers played a central role in tailoring risk communication to local needs and building trust [9]. These examples highlight that community-based actors can be effective vehicles for dialogue, particularly where formal structures for participatory communication are weak or absent. Hotlines represent another underutilized tool for promoting dialogue. Free of charge telephone hotlines, although mentioned only briefly in GMS contexts, provided important channels for communities to seek information, voice concerns, and access medical assistance in several African countries [9]. Their absence in the GMS could be a missed opportunity to facilitate two-way communication at scale. Expanding such mechanisms in future health emergencies could help bridge the gap between centralized systems and the need for localized, responsive engagement.

### Ethical considerations of punitive measures

Vietnam’s approach to pandemic control relied heavily on punitive enforcement, including fines and arrests for non-compliance with public health measures [24]. These actions may have contributed to high rates of compliance and low transmission in the short term, but they raise important ethical and governance concerns. Evidence from pandemic governance research shows that coercive strategies, while effective in generating rapid behavioural change, can undermine public trust in state institutions and reduce willingness to cooperate in future crises [43]. In this sense, coercion risks delivering compliance at the expense of resilience. In South Korea, rather than punitive enforcement, compliance was largely achieved through transparent communication, extensive testing, and community-driven initiatives, illustrating that trust and participation can be mobilised as alternatives to sanctions [41]. The Vietnamese government’s simultaneous suppression of dissenting voices may have further weakened long-term trust. By penalising critics and limiting opportunities for public dialogue, authorities risked narrowing the space for civic engagement and debate, both of which are vital for adaptive and accountable crisis governance [24]. Studies on risk governance emphasise that trust is most effectively built when governments communicate openly, acknowledge uncertainties, and incorporate feedback, rather than relying solely on top-down enforcement [24]. In the Vietnamese context, the heavy reliance on penalties may have forced outward compliance but concealed underlying discontent, raising questions about whether the model is sustainable or transferable to future health emergencies.

### Lessons for future preparedness

The GMS experience highlights the importance of tailoring RCCE strategies to sociocultural contexts while prioritizing consistency in messaging and inclusivity. Effective risk communication requires more than disseminating information—it necessitates dialogue, trust-building, and targeted outreach to marginalized groups. The effectiveness of Thailand’s VHVs illustrates the potential of leveraging community networks, yet their limitations in reaching all segments of society — particularly undocumented migrant workers or those in precarious employment — demonstrates gaps that are not unique to COVID-19. Similar barriers have been identified in relation to HIV [52], tuberculosis [53], and other health issues [54], suggesting that preparedness efforts should anticipate these challenges and design mechanisms in advance, rather than improvising during outbreaks and relying already overburdened community based care providers. Future efforts should focus on addressing systemic barriers to communication, integrating two-way communication mechanisms, such as hotlines and interactive media. Additionally, the ethical implications of punitive enforcement measures warrant careful consideration to balance public safety with community trust. While Vietnam and Thailand’s responses were praised for their effectiveness in minimizing COVID-19 mortality, the reliance on authoritarian governance and suppression of dissent raises questions about the sustainability and equity of such approaches. Comparisons with decentralized, participatory models in other regions highlight the need for an approach that acts on previously built compliance and trust. As the GMS prepares for future health emergencies, a more participatory and inclusive model of RCCE will be essential to achieving equitable and resilient outcomes.

### Limitations

This review provides a timely synthesis of RCCE initiatives in a high-priority region for emerging infectious diseases. However, its reliance on English-language articles may have excluded relevant studies published in local languages. Additionally, the rapid review methodology necessitated trade-offs between comprehensiveness and timeliness. Given the rapid evolution of COVID-19 pandemic, preprints and grey literature played a significant role in disseminating timely research. However, to maintain rigor and reliability, we prioritized peer-reviewed studies in this review. Preprints were only considered if they provided unique, high-quality insights into RCCE and were later validated by subsequent peer-reviewed literature. This approach helped balance the need for timely evidence while ensuring methodological robustness. The exclusion of articles that focused primarily on the consequences of the pandemic may have introduced a limitation as pandemic consequences are often interlinked with communication strategies. By omitting studies that emphasized consequences, we may underrepresent the indirect or negative effects of RCCE, and risk presenting an overly positive or neutral picture of communication strategies. Future research that explicitly integrates RCCE approaches with analyses of pandemic consequences would provide a more holistic understanding of both the effectiveness and the unintended impacts of communication in health emergencies.

To refine the scope and relevance of the review we only focused on databases that provided more targeted coverage of relevant literature (e.g., medicine, public health, or environmental science), compared to Scopus, which is broader and includes non-peer-reviewed sources like conference proceedings. A question arose on defining the population and including the correct terms for different regional groups. While searching in the databases, we noticed that even though some ethnic groups were mentioned, the articles often only mentioned the country. Therefore, we only included terminology that refers to the country and not specific ethnic groups, such as the Khmer in Cambodia. This is contained in the country’s names with truncation, like Cambodia*, Vietnam*, Myanma*, and written out like Thai, Thais, and Laotian*. We did not include the names of the groups living in “Yunnan province” or “Guangxi”. We included Mesh-terms for the countries in the search in PubMed, and some of the entry terms of the Mesh included words on ethnic groups and people living in the countries.

## Conclusion

The GMS implemented several initiatives related to RCCE during COVID-19. Vietnam demonstrated innovative and centralized communication approaches, using viral songs, slogans, and multimedia campaigns to effectively engage its population, while Thailand relied on its extensive network of VHVs to reach underserved communities, providing health education and monitoring. The pandemic’s second wave highlighted complex challenges relating to misinformation, access to hard-to-reach populations and inconsistent participatory and punitive approaches. These findings demonstrate the need for adaptable and inclusive strategies that integrate multi-channel communication, allow fortwo-way dialogue, and prioritize trust-building, particularly among vulnerable populations. 

## Data Availability

Not applicable.
